# Prolonged FOS activity disrupts a global myogenic transcriptional program by altering 3D chromatin architecture in primary muscle progenitor cells

**DOI:** 10.1186/s13395-022-00303-x

**Published:** 2022-08-15

**Authors:** A. Rasim Barutcu, Gabriel Elizalde, Alfredo E. Gonzalez, Kartik Soni, John L. Rinn, Amy J. Wagers, Albert E. Almada

**Affiliations:** 1 Department of Stem Cell and Regenerative Biology, Harvard University, Cambridge, MA USA; 2 Present address: Donnelly Centre, University of Toronto, Toronto, ON Canada; 3 Department of Orthopaedic Surgery, University of Southern California, Los Angeles, CA USA; 4 Department of Stem Cell Biology and Regenerative Medicine, University of Southern California, Los Angeles, CA USA; 5 Present address: BioFrontiers and Department of Biochemistry, University of Colorado Boulder, Boulder, CO 80303 USA

**Keywords:** Muscle satellite cells, Muscle progenitor cells, FOS, AP-1, Myogenic differentiation, Hi-C, Topologically associated domains (TADs), gene loops

## Abstract

**Background:**

The AP-1 transcription factor, FBJ osteosarcoma oncogene (FOS), is induced in adult muscle satellite cells (SCs) within hours following muscle damage and is required for effective stem cell activation and muscle repair. However, why FOS is rapidly downregulated before SCs enter cell cycle as progenitor cells (i.e., transiently expressed) remains unclear. Further, whether boosting FOS levels in the proliferating progeny of SCs can enhance their myogenic properties needs further evaluation.

**Methods:**

We established an inducible, FOS expression system to evaluate the impact of persistent FOS activity in muscle progenitor cells ex vivo*.* We performed various assays to measure cellular proliferation and differentiation, as well as uncover changes in RNA levels and three-dimensional (3D) chromatin interactions.

**Results:**

Persistent FOS activity in primary muscle progenitor cells severely antagonizes their ability to differentiate and form myotubes within the first 2 weeks in culture. RNA-seq analysis revealed that ectopic FOS activity in muscle progenitor cells suppressed a global pro-myogenic transcriptional program, while activating a stress-induced, mitogen-activated protein kinase (MAPK) transcriptional signature. Additionally, we observed various FOS-dependent, chromosomal re-organization events in A/B compartments, topologically associated domains (TADs), and genomic loops near FOS-regulated genes.

**Conclusions:**

Our results suggest that elevated FOS activity in recently activated muscle progenitor cells perturbs cellular differentiation by altering the 3D chromosome organization near critical pro-myogenic genes. This work highlights the crucial importance of tightly controlling FOS expression in the muscle lineage and suggests that in states of chronic stress or disease, persistent FOS activity in muscle precursor cells may disrupt the muscle-forming process.

**Supplementary Information:**

The online version contains supplementary material available at 10.1186/s13395-022-00303-x.

## Background

Adult skeletal muscle is one of the few tissues in mammals endowed with a remarkable ability to regenerate after injury. Muscle-specific stem cells, commonly referred to as satellite cells (SCs), are the key cellular source that drives the growth and repair of postnatal skeletal muscle [1, 2]. SCs reside under the basal lamina of myofibers, where they exist in a quiescent (non-dividing) state in unperturbed muscle. In response to myofiber destruction, SCs activate and migrate to sites of damage, proliferate to expand the progenitor cell pool, and further differentiate and fuse with existing myofibers to restore the muscle tissue to its original state [3, 4].

Recent work has shown that FOS/AP-1 is transiently induced in SCs within hours following muscle trauma [5–10]. In addition, it was found that FOS-expressing SCs display enhanced regenerative properties including rapid entry into cell cycle, efficient expansion of the stem/progenitor cell pool, and effective regeneration of skeletal muscle after injury [9]. These data suggest that FOS is not simply a marker of “early” SC activation but may also be involved in programming a “regenerative” gene network that instructs rapid muscle repair responses.

A question that has remained unresolved is why FOS is rapidly downregulated in adult SCs before they enter cell cycle as progenitor cells and further differentiate as myoblasts. Additionally, whether elevating FOS levels in the progeny of SCs can enhance their muscle-forming properties as it appears to do when transiently expressed in “early” activated SCs in vivo needs further investigation [9]. Indeed, several decades ago, multiple groups showed that transient transfection of *Fos* can disrupt *MyoD* and *MyoG* transcriptional activity from a muscle creatine kinase (*MCK*) reporter plasmid [11], as well as partially disrupt *MyHC* expression and myotube formation [12, 13]. However, these studies were performed in either non-muscle cells or in immortalized myoblast cell lines and not in primary muscle progenitor cells recently derived from SCs. Thus, the biological relevance of these observations regarding early muscle progenitor cell biology remains unclear.

The transcriptional activator FOS has been studied for over 40 years in various cell types and states [14], yet its molecular role in controlling gene expression in stem and progenitor cells after injury remains largely unexplored [15]. Recent technological advances in the post-genomic era has shown that DNA is folded into higher-order chromatin structures—such as A/B compartments, TADs, and gene loops—that dynamically arrange genes into transcriptionally active or repressive domains [16]. Considering that chromosomal interactions are critical for defining key stem and progenitor cell fates [17–19] and are often mis-regulated in disease [20–25], there is substantial interest to identify factors that regulate how the 3D genome is organized in the nucleus.

In this study, we established a lentiviral, doxycycline-inducible system to explore the effects of persistent FOS activity on primary muscle progenitor cell fate decisions. In summary, we found that prolonged FOS expression in SC-derived muscle progenitor cells has minimal impact on their proliferative status but severely blunts their terminal differentiation potential within the first few weeks in culture. We further demonstrate through an integrative approach, using RNA-sequencing (RNA-seq) coupled with high-throughput chromosome conformation capture (Hi-C) analysis, that persistent FOS activity in muscle progenitor cells suppress a global myogenic transcriptional program by mis-regulating the local 3D chromatin architecture. Collectively, this work highlights the complex properties of FOS/AP-1 within the muscle lineage, and further suggests that uncontrolled FOS activity in the “early” progeny of SCs (i.e., progenitor cells) may be detrimental to skeletal muscle repair.

## Results

### Doxycycline-inducible system to ectopically express FOS in muscle progenitor cells

**Fig. 1:**
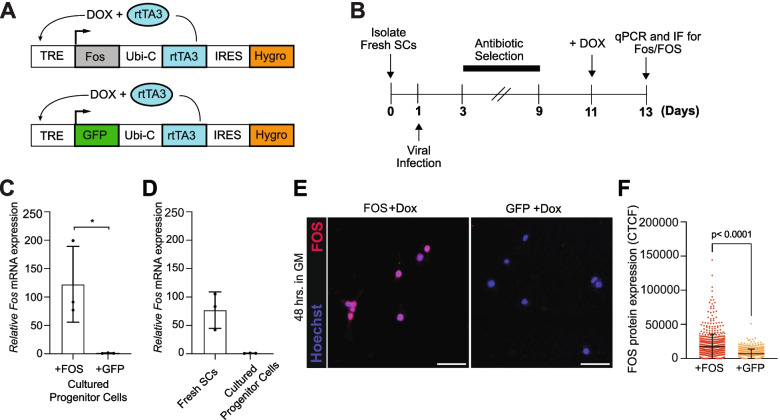
Establishing a doxycycline-inducible system to ectopically express FOS in muscle progenitor cells. **A** Schematic of the doxycycline-inducible lentiviral system to express FOS or GFP. The all-in-one vector contains the gene of interest, the rtTA, and a selection marker in a single expression vector. The Ubiquitin-C (Ubi-C) promoter drives the constitutive expression of the rtTA, and hygromycin via an internal IRES sequence. In the presence of DOX (1 μg/ml), rtTA becomes activated and binds to the TRE promoter to drive expression of *Fos* or *Gfp*. **B** Experimental design schematic. 3000 SCs were seeded into wells containing GM, infected with virus expressing FOS or GFP, selected with hygromycin (100 μg/ml) for 6 days, rested for 2 days, and then 4000 cells were re-seeded into wells containing GM supplemented with 1 μg/ml of DOX. **C** Relative expression of *Fos* mRNA normalized to GAPDH in pSLIK-*Fos* muscle progenitor cells relative to pSLIK-*Gfp* muscle progenitor cells after 48 h in GM supplemented with 1 μg/ml of DOX (*N* = cells from 3 mice). **D** Relative expression of *Fos* mRNA (normalized probe signal intensity, microarray data [9]) in freshly isolated SCs relative to 5-day cultured SCs. **E** 20× images of cultured muscle progenitor cells (FOS+DOX and GFP+DOX) stained for FOS (Red) or Hoechst (nuclei), showing FOS-positive Hoechst-positive nuclei in cells infected with pSLIK-*Fos* virus. Scale bar represents 50 μm. **F** Corrected total cell fluorescence (CTCF) of FOS protein in individual pSLIK-*Fos* and pSLIK-*Gfp* muscle progenitor cells quantified in **E** (*n* = 1480 (FOS) and 1012 (GFP) cells. **C** Mean comparisons using an unpaired, two-tailed, Student’s *t* test and **F** using Mann-Whitney *U* test. Data represents mean ± SD

To investigate the molecular and functional consequences of continuous *Fos* expression in primary muscle progenitor cells (i.e., progeny of SCs), we took advantage of a recently developed all-in-one lentiviral, doxycycline (DOX)-inducible gene expression system (pSLIK) [26]. In this system, the reverse tetracycline transactivator (rtTA), antibiotic selection marker (hygromycin), and the gene of interest are induced from a single expression cassette. For our studies, we cloned a *Fos* or *Gfp* cDNA into the pSLIK vector, so that in the presence of DOX, rtTA will activate the TRE promoter and drive the expression of either FOS or GFP (Fig. 1A). We determined that 1 μg/ml of DOX was the optimal concentration to add to the growth media (20% donor horse serum, 1% Glutamax, 1% Pen/Strep; 5 ng/mL of bFGF in F10 media, GM) that had no significant effect on cell morphology and expansion of non-infected muscle progenitor cells (Additional file 1: Figure S1A, B), but also maintained maximal induction of FOS protein in muscle progenitor cells infected with pSLIK-*Fos* virus (Additional file 1: Figure S1C, D). We note that most non-infected muscle progenitor cells were PAX7-positive and MYOG-negative after being cultured in GM for ~ 2 weeks (Additional file 1: Figure S1E, F).

After defining the optimal DOX concentration, we infected freshly isolated SCs with lentivirus containing *Fos* (pSLIK-*Fos*) or *Gfp* (pSLIK-*Gfp*) after 1 day in culture, selected each culture with hygromycin (starting on day 3) for 6 days and allowed the cells to recover for 2 days. Subsequently, we re-seeded 4000 pSLIK-*Fos* and 4000 pSLIK-*Gfp* muscle progenitor cells per well and then cultured each population in GM supplemented with DOX for 48 h (Fig. 1B). Under these conditions, we observed a substantial induction of *Fos* mRNA (~ 120-fold) in cultured pSLIK-*Fos* muscle progenitor cells to levels only moderately higher (~ 65-fold) than what is found physiologically in early-activated SCs recently isolated from uninjured skeletal muscle via fluorescence activated cell sorting (FACS) (Fig. 1C, D) [9]. Consistently, we also observed a rapid induction of FOS protein in pSLIK-*Fos* muscle progenitor cells but significantly lower signal in pSLIK-*Gfp* muscle progenitor cells (Fig. 1E, F). Additionally, pSLIK-*Fos* and pSLIK-*Gfp* cell populations express PAX7 and have minimal expression of MYOG (Additional file 1: Figure S1G). Together, we established an inducible FOS expression system that enables us to elevate *Fos* in the muscle lineage, and importantly, evaluate the impact of FOS activity on key muscle progenitor cell fate decisions.

### Persistent FOS expression in muscle progenitor cells disrupts myogenic differentiation

**Fig. 2:**
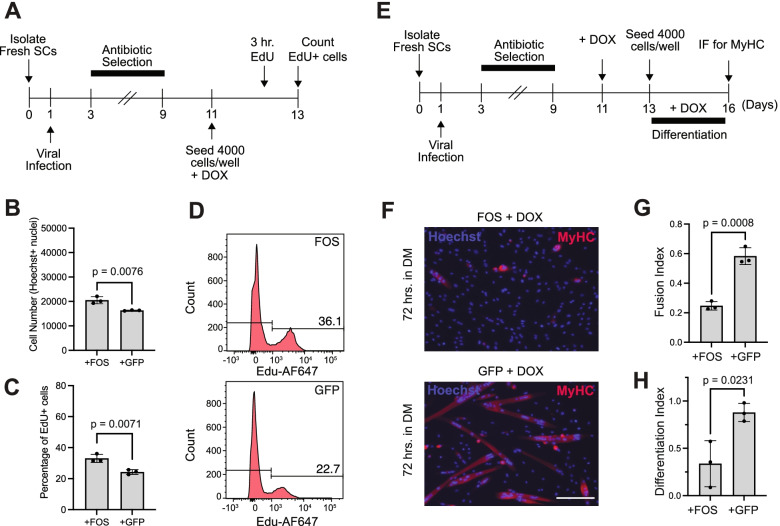
Persistent FOS expression in muscle progenitor cells disrupts myogenic differentiation. **A** Experimental design. Fresh SCs were isolated from skeletal muscle (see “Materials and methods” section), infected with either pSLIK-*Fos* or pSLIK-*Gfp* viral vectors 1 day after isolation, selected with hygromycin (100 μg/ml) for 6 days, and then re-seeded at 4000 cells per 96-well in GM supplemented with 1 μg/ml of DOX for 48 h. Three hours before the end-point, cultures were pulsed with EdU. **B** Total number of Hoechst+ cells per well after 48 h in GM supplemented with 1 μg/ml of DOX (*N* = cells from 3 mice). **C** Percentage of EdU+ cells after 48 h in GM supplemented with 1 μg/ml of DOX (*N* = cells from 3 mice). **D** Representative histogram showing EdU(−) cells and EdU(+) cells, as defined by a fluorescent-minus-one control to set the negative and positive gates in flow cytometry. **E** Experimental design. Fresh SCs were isolated from skeletal muscle, infected with either pSLIK-*Fos* or pSLIK-*Gfp* viral vectors 1 day after isolation, selected with hygromycin (100 μg/ml) for 6 days, expanded in GM supplemented with DOX (1 μg/ml) for 48 h, and then 4000 cells were seeded per 96-well in DM (2.5% Horse Serum in DMEM) supplemented with DOX (1 μg/ml) and cultured for 72 h. **F** 20× image of pSLIK-*Fos* or pSLIK-*Gfp* myogenic cultures after 72 h in DM showing Hoechst+ (blue) and MyHC+ (magenta) cells. Scale bar represents 100 μm. **G** Quantification of the fusion index (total number of Hoechst+ nuclei in MyHC+ myotubes divided by the total number Hoechst+ nuclei) in pSLIK-*Fos* or pSLIK-*Gfp* myogenic cultures (*N* = cells from 3 mice). **H** Quantification using a differentiation index (sum of the integrated intensity of all Hoechst+ objects within MyHC+ objects divided by the sum of the integrated intensity of all Hoechst+ objects) in pSLIK-*Fos* or pSLIK-*Gfp* myogenic cultures (*N* = cells from 3 mice). **B**, **C**, **G**, **H** Mean comparisons using an unpaired, two-tailed, Student’s *t* test

Muscle progenitor cells in culture will continue to proliferate in standard growth media (GM) or terminally differentiate into myotubes when switched into differentiation media (DM, 2% Horse Serum in DMEM). Thus, the cultured environment serves as a highly controllable ex vivo system to evaluate two of the most critical progenitor cell fate decisions—cell proliferation and differentiation. To determine how persistent FOS activity impacts muscle progenitor cell proliferation under standard growth conditions, we seeded 4000 pSLIK-*Fos* and 4000 pSLIK-*Gfp* transduced muscle progenitor cells into wells with GM and allowed both cell populations to expand for 48 h in the presence of DOX (Fig. 2A). Overall, we found that the mean number of Hoechst+ cells among pSLIK-*Fos* cells was 22,111 (± 1446) and for pSLIK-*Gfp*-cells it was 16,319 (± 174) after 48 h in culture. In agreement, the percentage of EdU+ cells in those wells initially seeded with pSLIK-*Fos* cells or pSLIK-*Gfp*-cells was 33.1% (± 2.6) and 24.3% (± 1.5) after 48 h in culture, respectively. These data suggest that persistent FOS expression in cultured primary muscle progenitor cells has a minor (20-30%) impact on cellular proliferation at the initial seeded density (Fig. 2B–D), an observation consistent with a recent study using C2C12 cells [27].

Next, to evaluate how persistent FOS activity in muscle progenitor cells impacts terminal myogenic differentiation, we expanded pSLIK-*Fos* and pSLIK-*Gfp* muscle progenitor cells in GM supplemented with DOX for 48 h (i.e., inducing FOS or GFP). Subsequently, we re-seeded 4000 cells from each group into wells containing DM supplemented with DOX and allowed the cells from each condition to differentiate for 72 h (Fig. 2E). Strikingly, we observed a ~ 2.5-fold decrease in terminal myogenic differentiation, as measured by manual quantification of the fusion index as well as using an automated image intensity-based terminal differentiation metric (see “Materials and methods,” section Fig. 2F–H). These results indicate that continuous FOS activity in SC-derived muscle progenitor cells severely blunts myogenic progression and differentiation within the first 2 weeks in culture.

### Prolonged FOS activity perturbs a myogenic gene expression program in muscle progenitor cells

**Fig. 3:**
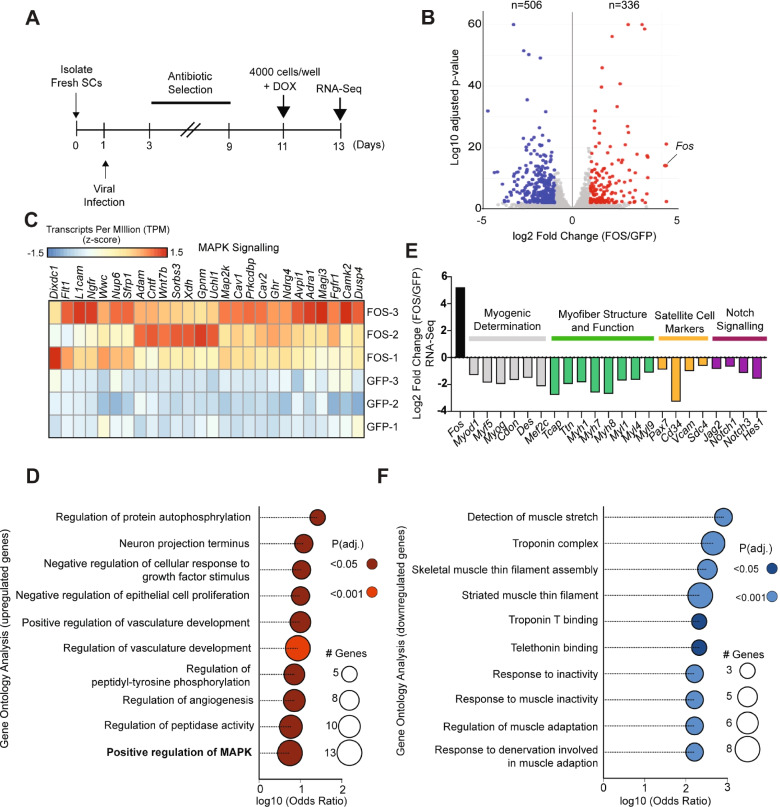
Prolonged FOS activity perturbs a myogenic gene expression program in muscle progenitor cells. **A** Experimental design. Fresh SCs were isolated from skeletal muscle (see “Materials and methods” section), infected with either pSLIK-*Fos* or pSLIK-*Gfp* viral vectors 1 day after isolation, selected with hygromycin (100 μg/ml) for 6 days, and then re-seeded at 4000 cells per 96-well in GM supplemented with DOX (1 μg/ml) and RNA was isolated for RNA-seq after 48 h (*n* = cells from 3 mice). **B** Scatterplot showing the average log10 normalized gene RNA-seq counts for pSLIK-*Fos* or pSLIK-*Gfp* muscle progenitor cells. The significantly up- and downregulated genes are labeled as red and blue, respectively. **C** Heatmap showing the scaled transcripts per million (TPM) for a cohort of MAPK Signaling genes that were enriched in pSLIK-*Fos* versus pSLIK-*Gfp* muscle progenitor cells. **D** Plot showing the significantly enriched Gene Ontology (GO) terms associated with genes upregulated in pSLIK-*Fos* muscle progenitor cells. **E** Highlighting the Log2 fold change (FC) between myogenic determination (grey), myofiber structure (green), SC marker (yellow), and notch signaling (purple) genes that were depleted in pSLIK-*Fos* versus pSLIK-*Gfp* muscle progenitor cells. **F** Plot showing the significantly enriched Gene Ontology (GO) terms associated with genes downregulated in pSLIK-*Fos* muscle progenitor cells

To uncover the molecular mechanisms by which prolonged *Fos* expression can disrupt myogenic progression, we performed poly(A) RNA-sequencing (RNA-seq) analysis in cultured muscle progenitor cells after expressing either FOS (pSLIK-*Fos*) or GFP (pSLIK-*Gfp*) for 48 h under standard growth conditions (Fig. 3A). Overall, we identified 336 up- and 506 downregulated genes (log2FC > 0.5 with an adjusted *p* value < 0.01) in pSLIK-*Fos* relative to pSLIK-*Gfp* muscle progenitor cells (Fig. 3B; Additional file 2: Table S1). We confirmed that *Fos* was among the most highly enriched mRNAs in pSLIK-*Fos* cells relative to pSLIK-*Gfp* cells (Fig. 3B). Based on Gene Ontology (GO) analysis, the other genes that were enriched in pSLIK-*Fos* muscle progenitor cells were associated with the MAPK signaling pathway and the regulation of angiogenesis, vascular development, metabolism, and cell communication (Fig. 3C, D; Additional files 2 and 3: Table S1 and 2).

More strikingly, we noticed that the mRNA for several of the canonical myogenic transcription factors (*Pax7*, *Myf5*, *MyoD*, *MyoG*), SC surface markers (*Sdc4*, *CD34*, *Vcam1*), and key Notch signaling members (*Jag2*, *Notch1/3*, *Hes1*) were substantially reduced in pSLIK-*Fos* muscle progenitors cells, most of which have key roles in maintaining SC identity and promoting muscle development and regeneration [4] (Fig. 3E). We also observed a broad down-regulation of genes encoding structural proteins that maintain the functional integrity of skeletal muscle in response to stress, and a substantial number of these genes cause various forms of muscular dystrophy when mutated in humans (i.e., *Tcap*, *Titin*, *Acta1*, *Myh3*, *Myh7*, *Myh8*, *Myl1*) [28] (Fig. 3E). Consistently, gene ontology (GO) analysis revealed that genes depleted in pSLIK-*Fos* muscle progenitor cells were associated with reinforcing muscle after stretching (i.e., telethonin binding) and the machinery involved in muscle contraction (i.e., Troponin complex, Troponin T binding) (Fig. 3F). FOS’s ability to suppress myogenic gene expression appears to be reversible, since we found that *MyoD*, *MyoG*, *Tcap*, and *Ttn* mRNAs are de-repressed within 3 days after DOX removal and concomitant with *Fos* returning to basal levels (Additional file 1: Figure S2). The mechanism of mRNA suppression mediated by FOS for some genes may include an H3K27me3-mediated process since ChIP-qPCR assays showed a 4-fold increase in H3K27me3 occupancy at the *MyoD* promoter (relative to an IgG control) in pSLIK-*Fos* cells but not in pSLIK-*Gfp* cells (Additional file 1: Figure S3). Collectively, these results suggest that prolonged FOS expression in cultured muscle progenitor cells disrupts a global transcriptional gene program that is required for myogenic lineage progression and proper muscle function, which at least for some genes is a reversible process and may include an H3K27me3-repressive mechanism.

### In situ hi-C analysis reveals higher-order chromatin structures in muscle progenitor cells

**Fig. 4:**
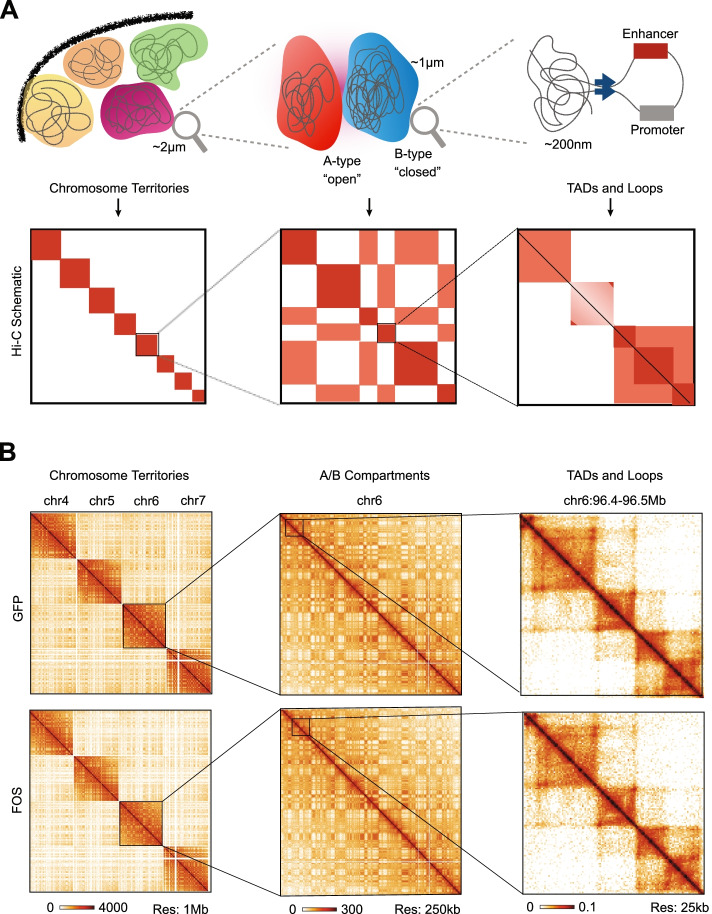
In situ Hi-C analysis reveals higher-order chromatin structures in muscle progenitor cells. **A** (Top) Schematic showing the nuclear organization of chromatin, displaying chromosome territories (~ 2 μm), A/B compartments (~ 1 μm), and TADs and Loops (~ 200 nm). (Bottom) Cartoon representation of Hi-C heatmaps corresponding to chromosome territories, A/B compartments, and TADs and loops as depicted in the top panel. **B** Representative Hi-C heatmaps of pSLIK-*Gfp* and pSLIK-*Fos* expressing muscle progenitor cells at several resolutions

Given that higher-order chromatin structure and its regulation plays a critical role in driving key progenitor cell fate decisions [17–19], we decided to explore the hypothesis that continuous FOS activity may alter how 3D chromatin is organized around critical myogenic genes. Over the last decade, Hi-C has emerged as the gold-standard method for mapping chromosomal interactions between local or distant DNA regions in a genome-wide manner [29]. Thus, in order to assess whether global chromatin organization is impacted by persistent FOS activity, we performed in situ Hi-C on cultured muscle progenitor cells expressing FOS (pSLIK-*Fos*) or GFP (pSLIK-*Gfp*) for 72 h in GM. Overall, we sequenced two biological replicates of Hi-C libraries from pSLIK-*Fos* and pSLIK-*Gfp* muscle progenitor cells to a depth of ~ 400 million reads per replicate. We confirmed that each replicate within a given condition showed a high degree of 1st eigenvector reproducibility (see “Materials and methods” section, Additional file 1: Figure S4). Overall, we identified many 3D chromosomal interactions at multiple scales including chromosome territories that are composed of open (i.e., transcriptionally active, A-type) and closed (i.e., transcriptionally inactive, B-type) genomic compartments at the megabase scale, as well as TADs and gene loops at the tens to hundreds of kilobase range (Fig. 4A, B).

### Continuous FOS activity leads to switching of A/B compartments near FOS regulated genes in muscle progenitor cells

**Fig. 5:**
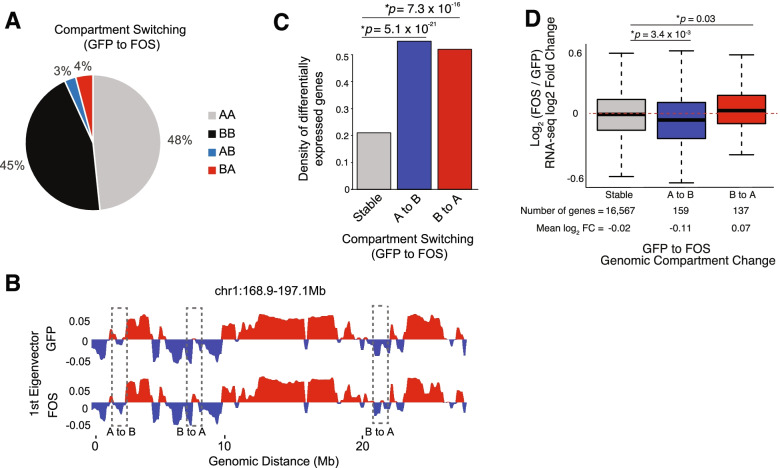
Continuous FOS activity leads to switching of A/B compartments near FOS regulated genes in muscle progenitor cells. **A** Pie chart showing the percentage of compartmental switching events between pSLIK-*Gfp* and pSLIK-*Fos* muscle progenitor cells. **B** Compartmentalization plot for chromosome 1 (168.9-197.1 megabases) showing the 1st eigenvector, where the positive values represent the open “A-type” and the negative values represent the closed “B-type” compartments, suggesting that while most of the A/B compartments in this genomic interval are unchanged, there are several examples of compartment switching events (dashed boxes) between the two conditions. **C** Bar plot showing the density of the normalized number of differentially expressed genes, based on RNA-seq data, at stable, or switched (i.e., A to B or B to A) compartmental regions (*p-value*: one-way ANOVA). **D** Box plot showing the average pSLIK-*Fos* vs. pSLIK-*Gfp* log2FC RNA-seq values for genes (outliers removed) within either stable compartments, or compartments that have switched from open to closed (A to B), and vice versa (B to A). *p value*: Wilcoxon rank-sum test

To evaluate whether A/B genome compartments at the megabase scale are altered in cultured muscle progenitor cells in the presence of continuous FOS activity, we generated “saddle plots” showing the average compartmentalization patterns in 250kb bins across the genome [30]. This analysis revealed nearly identical genomic compartment patterns between both conditions (Additional file 1: Figure S5A, C) [30], suggesting that persistent FOS activity in muscle progenitor cells does not result in large scale re-positioning of chromosomal A/B compartments within the nucleus. However, when we searched for genomic compartments that may have switched (i.e., A to B or B to A), we noticed that ~ 7% of the genome was switched in their A/B compartmentalization type (Fig. 5A). For example, we found that 3% of the genomic compartments showed a switch from an “open” A-type compartment in pSLIK-*Gfp* muscle progenitor cells to a “closed” B-type compartment in pSLIK-*Fos* muscle progenitor cells, whereas 4% of genomic compartments showed the opposite trend, a switch from a “closed” B-type compartment in pSLIK-*Gfp* muscle progenitor cells to an “open” A-type in pSLIK-*Fos* muscle progenitor cells (Fig. 5A, B). Importantly, the observed compartment switching events were found in both Hi-C biological replicates, indicating that these chromatin organizational events are highly reproducible in cultured muscle progenitor cells ectopically expressing FOS.

To establish whether FOS-mediated, A/B compartment switching events are associated with changes in RNA expression levels from neighboring genes, we performed an integrated analysis using our RNA-seq data and Hi-C interaction maps. We found a significantly higher number of differentially expressed genes in switched compartments when compared to the genes in stable compartments (*p* = 5.1 × 10^−21^ for A to B, *p* = 7.3 × 10^−16^ for B to A, one-way ANOVA) (Fig. 5C). Further, genes associated with compartments that switched from an open A-type to a closed B-type showed a significant decrease in mRNA expression (*p* = 3.4 × 10^−3^, Wilcoxon rank-sum test), whereas the genes located near compartments that switched from a closed B-type to an open A-type displayed a modest, but statistically significant increase in mRNA expression (*p* = 0.03, Wilcoxon rank-sum test) in pSLIK-*Fos* muscle progenitor cells (Fig. 5D). Importantly, gene ontology analysis of genes associated with A to B (opened to closed) switching events were involved in the regulation of muscle cell membrane potential, whereas genes associated with B to A (closed to open) switching events were linked with DNA accessibility including nucleosome regulation (Additional file 1: Figure S5D, E). Together, these results demonstrate that persistent FOS activity in muscle progenitor cells leads to a highly selective switching of A/B compartment events that correlate with the RNA expression of nearby FOS-regulated genes.

### Elevated FOS activity alters gene loops and TAD borders near myogenic genes in muscle progenitor cells

**Fig. 6:**
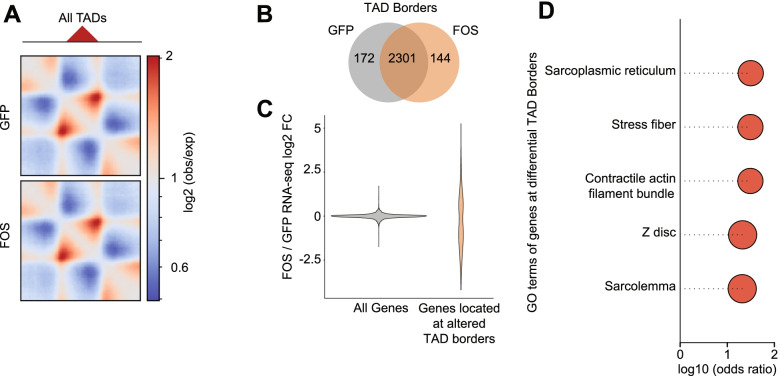
Elevated FOS activity alters TAD borders in muscle progenitor cells. **A** Meta-TAD plots, where the interaction profiles of all detected TADs (*n* = 2474 (pSLIK-*Gfp*), 2447 (pSLIK-*Fos*)) were scaled and superimposed on top of each other, showing a similar TAD formation in pSLIK-*Fos* relative to pSLIK-*Gfp* muscle progenitor cells. **B** Venn diagram showing the overlapping or differentially regulated (by at least 2 × 40 kb bins) TAD boundaries in pSLIK-*Gfp* and pSLIK-*Fos* muscle progenitor cells. **C** Violin plot showing pSLIK-*Fos* vs. pSLIK-*Gfp* RNA-seq log2FC values of genes located at overlapping or differentially regulated TAD boundaries. **D** Plot showing the GO enrichment of differentially expressed genes that are located at differentially regulated TAD boundaries. The adjusted *p* values for all GO enrichments are < 0.05

Within A/B genomic compartments—at a scale from tens to hundreds of kilobases—the genome is configured into contact domains such as TADs and loops, where genes located within a single TAD or gene loop are typically co-transcriptionally regulated [31–33]. To determine whether TADs are mis-regulated upon prolonged FOS expression, we defined the TAD boundaries in pSLIK-*Fos* and pSLIK-*Gfp* muscle progenitor cells using the insulation score method [34]. Although the strength and positioning of most of the TAD borders were unchanged (Fig. 6A; Additional file 1: Figure S6A, B), we identified a set of ~ 172 TAD boundaries that were weakened and 144 TAD boundaries that were strengthened in pSLIK-*Fos* relative to pSLIK-*Gfp* muscle progenitor cells (Fig. 6B). Interestingly, genes nearby these altered TAD boundaries displayed a more variable distribution of differential RNA expression levels (Fig. 6C) and were enriched for GO terms involved in muscle fiber contraction (e.g., sarcoplasmic reticulum, stress fiber and z-disc) (Fig. 6D).

**Fig. 7:**
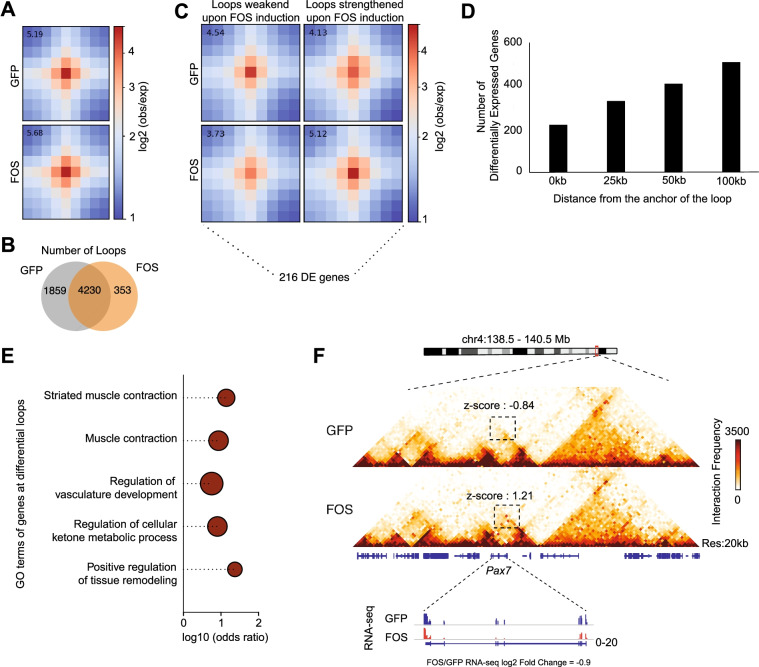
Elevated FOS activity alters gene loops near myogenic genes in muscle progenitor cells. **A** A meta plot showing the average loops (depicted as dots) in pSLIK-*Gfp* and pSLIK-*Fos* muscle progenitor cells. **B** Venn diagram showing the number of overlapping and differentially regulated loops in pSLIK-*Gfp* and pSLIK-*Fos* muscle progenitor cells. **C** Meta plots of all loops that have been lost or gained in pSLIK-*Fos* cells, showing loops weakened and strengthened in presence of FOS. **D** Bar plot showing the number of differentially expressed genes up to 100 kb around the differentially regulated loops at 25 kb intervals. Majority of the FOS-mediated differentially expressed genes are located within altered genomic loops. **E** Plot showing the GO term enrichment of differentially expressed genes within 25 kb distance of differentially regulated loops. **F** Heatmap at 20 kb resolution of GFP and FOS Hi-C datasets showing a strengthening of a loop formation at the *Pax7* gene locus, which is associated with a decrease in *Pax7* gene expression with a log fold-change value of − 0.9. The *z*-scores of the looping interaction are depicted on the figure

To evaluate how persistent FOS expression specifically impacts gene looping interactions across the genome [35–37], we calculated the number of differential looping interactions between pSLIK-*Fos and* pSLIK-*Gfp* muscle progenitor cells and discovered 353 loops that were gained and 1859 loops that were substantially lost upon continuous FOS activity (Additional file 1: Figure S7A, Fig. 7A–C). Interestingly, only 7% of these FOS-dependent gene loops overlap with a recent dataset representing MyoD-dependent gene loops in muscle cells (Additional file 1: Figure S7B) [38], suggesting that downregulation of *MyoD* mRNA, and thus, subsequent loss of MyoD-specific gene looping events alone cannot explain all the gene loops in pSLIK-*Fos* cells. In addition, among the differentially regulated gene loops in pSLIK-*Fos* relative to pSLIK-*Gfp* muscle progenitor cells, we found 216 (~ 25%) and 484 (> 60%) of the FOS-regulated differentially expressed genes (Fig. 3) located within 25 kb and 100 kb from the differentially regulated loops, respectively (Fig. 7D). Importantly, based on GO analysis, the neighboring genes near the differentially regulated loops are associated with muscle-specific functions (Fig. 7E). For example, we identified altered loops adjacent to the Myosin light chain 1 (*Myl1*) gene (Additional file 1: Figure S7A) and the canonical SC regulator gene, *Pax7* (Fig. 7F), both of which displayed altered mRNA expression in the presence of continuous FOS activity. Altogether, these data indicate that prolonged FOS activity in cultured muscle progenitor cells disrupts some TAD borders, but more drastically, alters the gene loop networks near crucial pro-myogenic genes.

## Discussion

FOS and other AP-1 family members regulate a multitude of cellular processes including proliferation, differentiation, and cell survival [39–41], suggesting that their specific function depends on the biological context and cellular state. While it was recently shown that transient FOS activity in early-activated SCs is crucial for eliciting an effective muscle regenerative response [5–9], the work presented here suggests that if FOS activity is not properly controlled in the progeny of SCs—as typically seen during normal adult muscle regeneration [9, 42, 43]—it may hinder the muscle reparative process. Using an inducible gene expression system in conjunction with genome-wide RNA-seq and Hi-C analysis, we confirmed the previously observed [11–13] antagonistic relationship between persistent FOS activity and myogenic progression using a more biologically relevant primary mouse muscle progenitor cell population. More importantly, we also significantly expand upon these prior data by cataloging the global transcriptional and 3D chromatin reorganization events that are altered as a consequence of prolonged FOS activity.

Through our transcriptome analysis, we reveal that continuous FOS activity disrupts a global pro-myogenic gene expression program that leads to a severe differentiation deficit in muscle progenitor cells. For example, we found reduced mRNA expression in all the classical muscle regulatory factors (MRFs) such as *Myf5*, *MyoD*, and *Myog*, and in genes required for myoblast fusion [44–47], muscle contraction, and for maintaining the structural integrity of skeletal muscle. Surprisingly, we also noticed down-regulation of key SC marker genes including *Pax7*, Syndecan-4 (*Sdc4*), *CD34*, *Vcam1*, and several members of the Notch signaling pathway (*Jag2*, *Notch1/3*, and *Hes1*), indicating that persistent FOS activity is unlikely to enhance progenitor cell performance given how crucial these genes are for normal myogenic function [48–51]. In contrast, genes involved in the MAPK signaling pathway were significantly enriched among the up-regulated genes in pSLIK-*Fos* muscle progenitor cells. Consistently, MAPK signaling is a known activator of FOS/AP-1 [52], and prior data also suggests that MAPK signaling promotes muscle differentiation and repair [53, 54]. Hence, whether the MAPK transcriptional signature represents a compensatory response by the progenitor cell to re-ignite the myogenic program, or rather hints towards an alternative MAPK/FOS-driven mechanism to repress myogenic activity upon chronic stimulation needs further investigation.

Regulating the 3D genome architecture (i.e., higher-order folding of chromatin) to facilitate interactions between distal regulatory enhancers and gene promoters [55–57] has emerged as a critical process driving key cell fate decisions including myogenic differentiation [19, 58–62]. In addition, long-range chromatin interactions and their associated gene-regulatory networks are often hijacked to advance disease [17, 20, 25, 59, 63–66]. For these reasons, there is a substantial need to identify the factors that regulate how chromatin folds around gene regulatory DNA elements [58, 61, 62, 67–71]. Recently, FOS/AP-1 was found to bind predominantly at gene regulatory enhancers—where it recruits tissue-specific transcription factors and chromatin modifiers—to facilitate gene activation [15, 72]. AP-1 has also been proposed to regulate long-range looping interactions at several individual gene loci [73–75] as well as in organizing multi-loop gene activation hubs [76]. However, experimental evidence demonstrating a role for FOS/AP-1 in directing chromatin interactions on a genome-wide scale has yet to be explored.

In our study, we report that persistent FOS activity in primary muscle progenitor cells results in a highly-specific misregulation of ~ 7% of A/B compartments (*n* = 191), 12% of TADs (*n* = 316), and 34% of individual looping interactions (*n* = 2212), all of which are located nearby FOS-regulated differentially expressed RNAs. Performing GO analysis on the genes proximal to the differentially regulated TADs and loops revealed associations with the sarcoplasmic reticulum, z-discs, and other mechanical units involved in skeletal muscle contraction. Thus, our data provides evidence that prolonged FOS activity in muscle progenitor cells disrupts the myogenic program, in part, by altering the local 3D chromatin organization near critical muscle-specific genes. Recently, *MyoD*, one of the genes down-regulated in pSLIK-*Fos* cells, has been shown to mediate 3D looping events in muscle cells, but only 7% of the MyoD-specific loops overlap with FOS-specific looping events in our study, indicating MyoD alone cannot explain most of our data (Additional file 1: Figure S7B). Future work is needed in order to further evaluate whether FOS is directly controlling the de novo formation or breakdown of gene loops and TADs, or instead is regulating the mRNA expression of additional downstream factors, that can directly reconfigure chromatin structure near pro-myogenic target genes [19, 58–60, 67, 77–80].

Considering that uncontrolled FOS activity appears to blunt myogenic progression so easily, it raises the question of whether this type of FOS misregulation ever occurs in the progeny of SCs in vivo. While our study is limited to *ex vivo* analysis, we uncovered several unexpected parallels in the phenotype and gene expression profile between adult muscle progenitor cells ectopically expressing FOS and dystrophic and aged SCs. First, previous work has shown that dystrophic and aged SCs have a deficiency in myogenic progression [81–83], similar to what we observe in muscle progenitor cells over-expressing FOS in culture. Second, we found reduced Notch and overactive MAPK signaling in pSLIK-*Fos* muscle progenitor cells, several pathways that are also coordinately misregulated in dystrophic [84–88] and aged [89–92] SCs. Further, the muscle SC pool in dystrophic and aged skeletal muscle progressively declines with age [93] and with the severity of muscular dystrophy [94], with a subset of SCs undergoing senescence [92] or transforming into fibro-adipogenic cell fates [95, 96]. Unexpectedly, we found that prolonged FOS activity in adult muscle progenitor cells led to the downregulation of multiple SC marker genes, indicating a vulnerability in maintaining the stem/progenitor cell identity. Moving forward, it will be important to evaluate whether an over-active or alternative FOS/MAPK pathway disrupts higher-order chromatin structures near critical pro-myogenic genes in aged [97] and dystrophic muscle stem/progenitor cells in vivo; thus, serving as one of the immediate molecular insults blocking proper stem/progenitor cell function in these debilitating conditions.

## Conclusions

Taken together, our study illustrates the importance of tightly controlling FOS expression during early myogenic commitment and differentiation and identifies FOS as a new transcriptional regulator that can rewire 3D chromosomal structures near crucial myogenic genes. Our work may suggest that in states of chronic stress or disease, over-active FOS may disrupt critical progenitor cell activities needed for muscle maintenance and efficient muscle repair.

## Materials and methods

### FACS-based isolation of primary muscle satellite cells

Primary muscle satellite cells (SCs) were isolated as previously reported in [98]. Briefly, hind-limb, abdominal, and tricep muscles were isolated from mice and digested in 0.2% collagenase type II (285 U/mg, Thermo Fisher, #17101015) in Dulbecco’s modified Eagle’s medium (DMEM, Gibco, #11965-092) for 90 min at 37 °C. Single fibers were separated from digested muscle by trituration and enriched through several gravitational sedimentation steps at 37 °C. Single fibers were digested in 0.0125% Collagenase type II/0.05% Dispase (1.81 U/mg, Thermo Fisher, #17105041) in F10 for 30 min at 37 °C to release mono-nuclear cells from the basal lamina of individual muscle fibers. The resulting cell suspension was spun (~ 500 rpm) to remove cellular debris, and then filtered through a 70-μm cell strainer. Cells were incubated on ice for 30 min with fluorescently conjugated antibodies to detect the following antigens: anti-Ly6A/E-APC or anti-Ly6A/E-PE (Sca-1) (1:200, BioLegend, RRID:AB_756197), anti-CD31-APC (1:200, BioLegend, RRID:AB_312917), anti-CD45-APC (1:200, BioLegend, RRID:AB_312977), CD11b-APC (Mac-1, BioLegend, RRID:AB_312795) (1:200), CD29-APC-Cy7 (β1-Integrin, BioLegend, RRID:AB_2128076) (1:200), CD184-Biotin (CXCR4, RRID:AB_2650787) (1:100). For CXCR4 detection, a secondary antibody, Streptavidin-PeCy7 (1:200, BioLegend, RRID: AB_2737413), was incubated with the relevant samples for 20 min on ice. Stained cells were analyzed by FACS using the BD FACSAria III cell sorter. After gating on physical parameters and live cells (Propidium Iodide-negative/Calcein Blue-positive) [98], SCs were defined as the Sca1^−^; CD45^−^; CD11b^−^; Ter119^−^; CD31^−^; CD29^+^ (β1-Integrin); CD184^+^ (CXCR4) cell population.

### Culture of primary satellite cells and C2C12 myoblast cells

Freshly isolated SCs were sorted into pre-coated, 96-well plates containing standard Growth Media (GM) consisting of 20% donor horse serum (DHS, Atlanta Biologicals), 1% penicillin/streptomycin (Gibco), 1% GlutaMax (Gibco), and 5 ng/mL of bFGF (Sigma) in F10 media. Plates were pre-coated for 24 h with a solution containing collagen type 1 (1 μg/ml final, MilliporeSigma, #C7661) and Laminin (10 μg/ml, Invitrogen, #23017-015) in PBS. GM was refreshed every 48-72 h, and cells were passaged (harvested) by removing half the volume of GM and adding back 5 mM EDTA (to achieve a 2.5-mM final concentration), incubating for 20 min at 37 °C, spinning at 1200 RPM, resuspending in fresh GM, counting, and seeding cells to new wells at desired densities. C2C12 cell lines (C2C12 ATCC CRL-1772) were purchased from ATCC and cultured according to the manufacturer’s recommendations.

### Generation of lentivirus

To produce lentivirus that express FOS or GFP in a Doxycycline (DOX)-inducible manner, we cloned *Fos* and *eGFP* cDNA into the MCS (i.e., Not1 and Kpn1 sites) of the entry vector, pEN_TTmcs (Addgene, #25755), and then recombined the relevant portions of this plasmid into the pSLIK-Hygro (Addgene, #25737) destination vector using Gateways LR Clonase II kit. HEK293T cells were seeded at 400,000 cells per 10 cm dish in DMEM supplemented with 10% FBS for 24 h prior to transfection. Briefly, we co-transfected 10 μg of pSLIK plasmid, 7.5 μg of each packaging plasmids pMDLg/pRRE (Addgene, #12251) and pRSVREV (Addgene, #12253), and 5 μg VSV-G (Addgene, #8454) envelope plasmid using Invitrogen’s Lipofectamine 2000 reagent into 293T cells. Viral supernatant was collected 72-h after transfection, concentrated using PEG-it virus precipitation solution (System Biosciences), resuspended in 200 μL of myogenic Growth Media (GM) supplemented with 10 mM Hepes buffer (virus mix), and aliquoted and stored at − 80 °C.

### Infection of fresh satellite cells

We titrated virus to determine the least toxic amount of virus that infected the most cultured stem/progenitor cells, as determined by hygromycin selection for 5-6 days. In brief, 3000 freshly isolated SCs were sorted into 96-well plates with 100 μL of GM media (100 μL), and within 1 h, 100 μL of a viral-media mix (consisting of 1/300 amount of virus and 8 μg/mL of Polybrene in GM) was added to each well of a 96-well plate (200 μL total). The plate was then covered with parafilm and incubated at 37 °C for 15 min, and then subsequently spun at 2000 RPM in a Beckman Coulter Allegra 6KR at 32 °C for 1.5 h. Virus was removed from the wells by extracting 100 μL of the viral-media mix and adding back 100 μL of fresh GM 8 consecutive times.

### Ex vivo proliferation and differentiation assays

Muscle progenitor cells were seeded at a density of 4000 cells per 96-well in GM and cultured either in the absence or presence of Dox (1 μg/mL). Muscle progenitor cell expansion was quantified by fixing the cell cultures in 4% PFA for 15 min, washing 3× with PBS, staining the nuclei with Hoechst dye, and counting the total Hoechst-positive nuclei using the Celigo high-throughput imaging platform (Nexcelcom) software. For measuring cell cycling kinetics directly, EdU (10 μM) was pulsed for 3 h in the cultures, and then samples were processed according to the manufacturer’s protocol (Click-IT EdU Alexa Fluor 647 Flow Cytometry, Invitrogen). We differentiated muscle progenitor cells by seeding 4000 cells per 96-well in DM (2.5% donor horse serum and 1% Penn/Step in DMEM) and cultured them in DOX (1 μg/ml, Sigma, #NDC-42806312-05) for 72 h. Fusion index was determined using manual quantification of multiple (> 4) 20× images covering the dish followed by evaluating the number of Hoechst-positive nuclei in myosin heavy chain (MyHC)+ cells over the total number of Hoechst-positive nuclei in the field. As an additional approach, we performed imaging and quantification using a Celigo imaging cytometer as previously reported [98]. Briefly, in multiple representative images for each condition, we defined the “differentiation index” as the sum of integrated intensity of Hoechst within all MyHC+ objects divided by the sum of the integrated intensity of all objects per well. This method largely recapitulates the results shown by manual counting of the fusion index (Fig. 2G, H).

### Immunofluorescence (IF) and immunohistochemistry (IHC)

IF was performed by fixing cells with 4% PFA for 15 min followed by 3 × 5 min. washes in PBST (0.1% Tween in PBS). Cells were permeabilized with 0.3% Triton-X in PBS for 20 min and blocked with 3% bovine serum albumin (BSA), 5% normal goat serum (NGS), 8% protein concentrate (Vector Labs, MOM-Immuno-Detection Kit), and 0.1% Triton-X in PBS for 1 h at room temperature. Cells were incubated with primary antibodies (1:1500 for anti-FOS, Abcam, RRID: AB_2106765; 1:250 for anti-Myosin, M4276, Sigma, RRID: AB_477190; 1:50 for anti-MyoG. BD Biosciences, 556358, RRID: AB_396383) O/N at 4 °C. Secondary antibody (Anti-mouse IgG, Abcam, 1:250, #A-21422) was added to 3% BSA in PBST and incubated with cells for 1 h at RT. Cells were washed 3× with PBST after each step. Hoechst dye was added into the final wash step for nuclei detection.

### Image acquisition and analysis

Cells in 96-well plates were imaged using a Zeiss LSM780 AxioObserver Z1 confocal microscope and a Plan-APO 20× 0.8 NA air objective. Images were saved as raw .lsm files and exported to FIJI(ImageJ) for analysis including maximum intensity projections, threshold adjustment and fluorescence intensity quantifications. After thresholding, FIJI’s Analyze Particles function was used for quantifying integrated cell fluorescence. Background fluorescence was measured in five unique and non-overlapping areas for every image and averaged for obtaining the average background fluorescence for a given image. Corrected total cell fluorescence (CTCF) was calculated using the formula CTCF = Integrated Density – (Area of selected cell × mean fluorescence of background readings). Scatter dot pots were generated using GraphPad Prism. Images acquired from three independent replicate cultures were used for quantification and statistics were performed using a two-tailed, non-parametric *t* test with Mann-Whitney correction, *p* values were calculated and indicated for each comparison.

### Protein analysis with WES (ProteinSimple)

Protein lysate was isolated from C2C12 cells using RIPA (ThermoFisher) buffer and quantified with a Bradford Assay (Bio-Rad) according to the manufacturer’s protocol. Antibodies, sample protein lysates, and other necessary reagents were added to a 25–110 Kd Chip, and subsequently loaded onto the WES detection system. Resulting data was analyzed using Compass software.

### RNA isolation and qPCR gene expression analysis

RNA was isolated using Qiagen’s RNeasy Micro Kit (74004, Qiagen) and processed according to the manufacturer’s protocol. We generated cDNA using the SuperScript IV VILO cDNA synthesis Kit (11756050, Thermo Fisher), and quantitative PCR of target genes was performed with the SybrGreen Master Mix reagent. qPCR plates were run and analyzed on an ABI 7500 platform or QuantStudio 6 Flex (Thermo Fisher) qRT-PCR machine. Relative expression and Fold-Difference was determined using the delta-delta-Ct method.

### ChIP-qPCR assays

Primary satellite cells were sorted into 96 well plates and infected with either pSLIK-*Fos* or pSLIK-*Gfp* (1:300 viral titration) virus 1 day after isolation. Infected cells were selected for 6 days with hygromycin (100 μg/ml) and then expanded for 3 weeks in growth media to generate 6 × 15 cm plates for pSLIK-*Fos* and 6 × 15 cm plates for pSLIK-*Gfp* muscle progenitor cells, yielding ~ 2 million cells per plate. Muscle progenitor cells were cultured in GM supplemented with doxycycline (DOX, 1 μg/mL) for 48 h to induce FOS or GFP expression. ChIP was performed using the Diagenode iDeal ChIP-qPCR kit (C01010180, Diagenode). In brief, at the end of the 48-h time-point, cells were harvested and resuspended in GM and then crosslinking buffer (including 1% Formaldehyde) was added to a proportion of 1:10 and then tubes were rotated at 20 rpm for 8 min at RT. The crosslinking reaction was quenched with 0.125 M glycine. Sonication of chromatin was performed with 10 cycles of 30 s “on” and 30 s “off” at 4 °C on a Pico Bioruptor (Diagenode). Two micrograms of an H3K27Me3-specific antibody (C15410195, Diagenode) or 1 μg of an Immunoglobulin G (IgG) only antibody (C15410206, Diagenode) was coupled to 30 μL of protein-A coated magnetic beads for every IP reaction and then incubated for 3 h at 4 °C while rotating at 15 rpm. Input samples were prepared with 1% of sheared chromatin volume from either pSLIK-*Fos* or pSLIK-*Gfp* cell conditions. For every IP, sheared chromatin was added to conjugated antibody-magnetic beads and incubated overnight at 4 °C while rotating at 15 rpm. IP’d chromatin was resuspended in 100 μL of DIB buffer and stored at – 20 °C or − 80 °C. We designed primers targeting the first 500 bases upstream of the gene TSS for *MyoD* and *Ttn*, which typically coincides with H3K27me3 enriched promoter proximal regions in activated muscle satellite cells in vivo (Liu et al. 2013). BMP6 and intergenic region control primers were obtained from a previous study (Hathaway, et al. 2012) but validated along with *MyoD* and *Titin* to ensure each primer yielded a single band and was efficiently priming over a 500-fold dilution series in qPCR (90–110%). qPCR reactions were performed with 3 μL of IP’d DNA or 3 μL of 1% input using SYBR Green master mix reagent (A25742, Thermo Fisher) according to the manufacturer’s protocol. qPCR reactions were run on a StepOnePlus qPCR system (Thermo Fisher), with 1 cycle of 50 °C for 2 min, then 95 °C for 2 min, followed by 40 cycles of 95 °C for 15 s and 60 °C for 1 min with a standard melting curve cycle performed. Percent input of enrichment was calculated with the following formula: 2^^(Ct [Input] - log2(100)) - Ct [IP])^ × 100.

### RNA-seq

Total RNA was extracted from GFP or FOS-expressing primary muscle progenitor cells using TRIzol followed by RNeasy Mini Qiagen extraction kit according to the manufacturer’s protocol. RNA-seq libraries were generated using the SMART-Seq ultra low RNA input kit for sequencing coupled with the Nextera XT DNA library kit and then sequenced on the Illumina HiSeq2500 platform. RNA-seq analysis (polyA) was performed by filtering and mapping the reads by Bowtie 2 [99] (mm9) and quantifying the transcripts by RSEM v1.2.29 [100]. Differential gene expression was calculated using DESeq2 by removing genes with a gene count < 10, and by using the mean value of gene-wise dispersion estimates. We defined differentially expressed genes as being changed > 0.5 log2 fold change in expression with an adjusted *p* value < 0.01 using the DEbrowser package [101]. Gene ontology (GO) analysis was performed using the FuncAssociate software version 3.0 [102]. Lower-level GO terms with > 1000 genes were not included in the analysis.

### Generation of hi-C libraries

In situ Hi-C libraries were generated using the *HindIII* restriction enzyme [103, 104]. Briefly, ~ 25 million cells were crosslinked with 1% formaldehyde for 10 min at room temperature. Then, the chromatin was extracted, digested with *HindIII,* end-labelled with biotin-14-dCTP, followed by in situ ligation (EL0011, Thermo Fisher). After DNA extraction, biotin was removed from unligated ends, and the sample was sheared using a Covaris S220 instrument (100–500 bp range) as previously described [104]. After A-tailing, biotin pull-down, and adapter ligation, we performed paired-end sequencing on Illumina’s HiSeq 2000 instrument. Each Hi-C library was generated with two biological replicates and sequenced to an average depth of ~ 400 million reads per replicate for each condition.

### Analysis of hi-C datasets

The resulting Hi-C sequencing reads were mapped (mm9), filtered, corrected, and binned using the HiC-Pro software v2.8 [105]. There was a high first eigenvector correlation among all the Hi-C biological replicates (average Pearson’s correlation *R*^2^ > 0.9), indicating the high quality and reproducibility of the datasets. Thus, we pooled all biological replicates for each condition and mapped, filtered, corrected, and binned them as a single Hi-C dataset for all subsequent analyses. Genomic A/B compartments and TADs (insulation method) were defined and visualized using the cworld, HiGlass, and Juicebox toolkits (cworld toolkit codes available at: https://github.com/dekkerlab/cworld-dekker), [106–108]. The saddle, meta-TAD and the meta-loop plots were generated using the cooltools package [109, 110]. Compartment switching analysis was performed as previously described [63]. Differentially regulated looping interactions were determined using the *hiccups* algorithm from the juicer toolkit [106].

## Supplementary Information

**Additional file 1: Supplementary Figure S1.** Related to Figure 1. Validation and characterizing of our DOX-Inducible system for manipulating FOS expression in Muscle Progenitor Cells Ex Vivo. (A) Representative images of cultured muscle progenitor cells in GM supplemented with increasing amounts of Doxycycline (0, 0.005 μg/ml, 0.01 μg/ml, 0.05 μg/ml, 0.1 μg/ml, 0.5 μg/ml, 1 μg/ml, 2.5 μg/ml, 5 μg/ml) for 48 hours in culture. Scale bars represent 50 microns. (B) Quantification of the total number of Hoechst+ cells after 48 hours in GM supplemented with the indicated concentration of DOX (n=cells from 3mice). (C) Experimental Flowchart for detecting FOS protein (sc-7292) using ProteinSimple WES platform and analysis using the COMPASS software. (D) Virtual bands showing gradual increase of FOS protein with increasing amounts of DOX. Raw area of signal is shown for loading control (GAPDH) and FOS. FOS signal normalized to GAPDH is displayed, highlighting that 1 ug/ml of DOX was the lowest concentration that gave the maximal induction of FOS protein. (E) 20X images of two-week cultured muscle progenitor cells grown in GM for 48 hours or differentiated in DM for 72 hours and stained for PAX7 and MyoG. Nuclei stained with DAPI. Scale bar represents 50 microns. (F) Corrected total cell fluorescence (CTCF) for PAX7 (left) and MYOG (right) quantified in (E). n= 98-4184 cells (PAX7) and n= 2169-46118 cells (MYOG). (G) Corrected total cell fluorescence (CTCF) for PAX7 (left) and MYOG (right) in two-week cultured pSLIK-*Fos* and pSLIK-*Gfp* muscle progenitor cells. n=2295-2816 (PAX7) and n=1432-3779 (MYOG). Mean comparisons using One-way ANOVA with post-hoc Tukey test (B) and Mann Whitney U-test (F, G). **Supplementary Figure S2.** Related to Figure 3. FOS-dependent suppression of myogenic genes in muscle progenitor cells is reversible upon DOX removal. pSLIK-*Fos* cells were treated with 1 ug/ml DOX for 72-hours and then chased with media devoid of DOX for 3 and 5 days followed by RT-qPCR. Mean relative mRNA expression (+/-SD) and normalized (to *Gapdh*) for *Fos, MyoD*, *MyoG*, *Tcap*, and *Ttn*. Data shows that upon removal of DOX, mRNA expression for *MyoD*, *MyoG*, *Tcap*, and *Ttn* are de-repressed concomitant with FOS returning to basal levels. Mean comparisons were performed with a one-way ANOVA with post hoc Tukey test. N= 5-6 replicate cultures from cells pooled from 4 mice. **Supplementary Figure S3.** Related to Figure 3. Chromatin Immunoprecipitation (ChIP)-qPCR analysis for H3K27Me3 occupancy at the promoter region of *Bmp6*, an intergenic region, *MyoD,* and *Ttn* in pSLIK-*Fos* and pSLIK-*Gfp* cultured muscle progenitor cells. (A) Experimental design: Primary satellite cells were infected with virus expressing pSLIK-*Fos* or pSLIK-*Gfp* constructs, selected with 100 μg/mL of hygromycin for 6 days, and expanded for approximately 3 weeks to generate enough cells for ChIP-qPCR. Once at the desired cell density, pSLIK-*Fos* and pSLIK-*Gfp* cells were cultured in GM-supplemented with 1μg/mL doxycycline (DOX) for 48 hours. Chromatin was IP’d using an H3K27Me3- or immunoglobulin G (IgG)-specific antibodies followed by qPCR. Plot shows mean percent of input (+/- SD) for each amplicon targeting the first 500bps upstream of the TSS for *Bmp6* and an intergenic region (left) and *MyoD* and *Ttn* (right). Location of primers relative to gene TSS is shown in schematic above each plot. Statistical comparisons were performed with two-way ANOVA with post hoc Holm-Sidak test. N= 8-9 replicate IPs were performed on cells pooled from 4 mice. **Supplementary Figure S4.** Related to Figure 4. Quality Metrics of Hi-C Datasets. (A) Pairwise comparison of all Hi-C heatmaps for each replicate of pSLIK-*Gfp* and pSLIK-*Fos* datasets showing all the chromosomes and the *inter*-chromosomal interactions. (B) Scaling plot showing the interaction frequency as a function of genomic distance, demonstrating that pSLIK-*Fos* and pSLIK-*Gfp* muscle progenitor cells have similar rates of decay of interaction frequency. (C) Representative higher resolution images of Hi-C datasets of pSLIK-*Gfp* and pSLIK-*Fos* muscle progenitor cells. **Supplementary Figure S5.** Related to Figure 5. Compartment Analysis of Hi-C Data. (A-B) Heatmap for the pSLIK-*Fos* and pSLIK-*Gfp* cells showing the compartment strength, quantified by plotting interaction frequencies in 250kb bins arranged by their values along the first eigenvector (PC1/EV1) to obtain compartmentalization saddle plots. The average interaction frequencies of the observed / expected interactions between pairs of loci (250kb bins) were calculated and arranged by their compartment signal (1^st^ eigenvector value) for pSLIK-*Fos* and pSLIK-*Gfp* muscle progenitor cells. In these plots the upper left quadrant represents B-B interactions, and the lower right corner represents A-A interactions. (C) Comparison of the *intra-* and *inter*-compartmental interaction frequencies indicates that FOS induction (i.e., pSLIK-*Fos* cells*)* results in significantly higher interactions within the A-type and B-type compartments (*p* < 2.2 x 10^-16^, Wilcoxon rank-sum test). In contrast, the *inter*-compartmental (between the A-type and B-type compartments) interactions were significantly decreased (*p* < 2.2 x 10^-16^, Wilcoxon rank-sum test) in pSLIK-*Fos* cells when compared to pSLIK-*Gfp* cells. (D) Gene ontology terms of differentially expressed transcripts whose coding regions have switched from open (A-type) to closed (B-type) compartmentalization. (E). Gene ontology terms of differentially expressed transcripts whose coding regions have switched from closed (B-type) to open (A-type) compartmentalization. **Supplementary Figure S6.** Related to Figure 6. Analysis of TAD boundaries. (A) A representative Hi-C heatmap at 40kb resolution for a 10 megabase genomic region (chr1:186,000,000-196,000,000) with insulation plots depicted on the bottom showing that most TADs are stable in pSLIK-*Fos* cells relative to pSLIK-*Gfp* cells. (B) Violin plot demonstrating similar TAD boundary strengths between pSLIK-*Gfp* and pSLIK-*Fos* datasets (*p-value:* Wilcoxon rank-sum test). **Supplementary Figure S7.** Related to Figure 6. Analysis of looping events. (A) Hi-C heatmap of pSLIK-*Gfp* and pSLIK-*Fos* datasets showing a reduction of a loop formation at the *Myl1* gene locus, which is associated with a decrease in *Myl1* gene expression (pSLIK-*Fos* vs. pSLIK-*Gfp* log2FC = -1.7). The z-scores of the looping interaction are depicted on the figure. (B) Overlap in MyoD-specific looping events in [38] that overlaps with FOS-specific looping events identified in this study.

**Additional file 2: Supplementary Table S1.** Differentially expressed genes in pSLIK-FOS expressing muscle progenitor cells relative to pSLIK-GFP expressing muscle progenitor cells (Related to Fig. 3).

**Additional file 3: Supplementary Table S2.** Gene ontology terms of up- and down-regulated genes in pSLIK-FOS expressing muscle progenitor cells relative to pSLIK-GFP expressing muscle progenitor cells (Related to Fig. 3).

## Data Availability

The datasets supporting the conclusions of this article are available in the GEO repository GSE166241 (https://www.ncbi.nlm.nih.gov/geo/query/acc.cgi?acc=GSE166241).
