# Supplementary material for: Prolonged FOS activity disrupts a global myogenic transcriptional program by altering 3D chromatin architecture in primary muscle progenitor cells
Source: Skelet Muscle. 2022 Aug 15;12:20. doi: 10.1186/s13395-022-00303-x (PMC9377060; doi:10.1186/s13395-022-00303-x)

Supplementary Figure S1

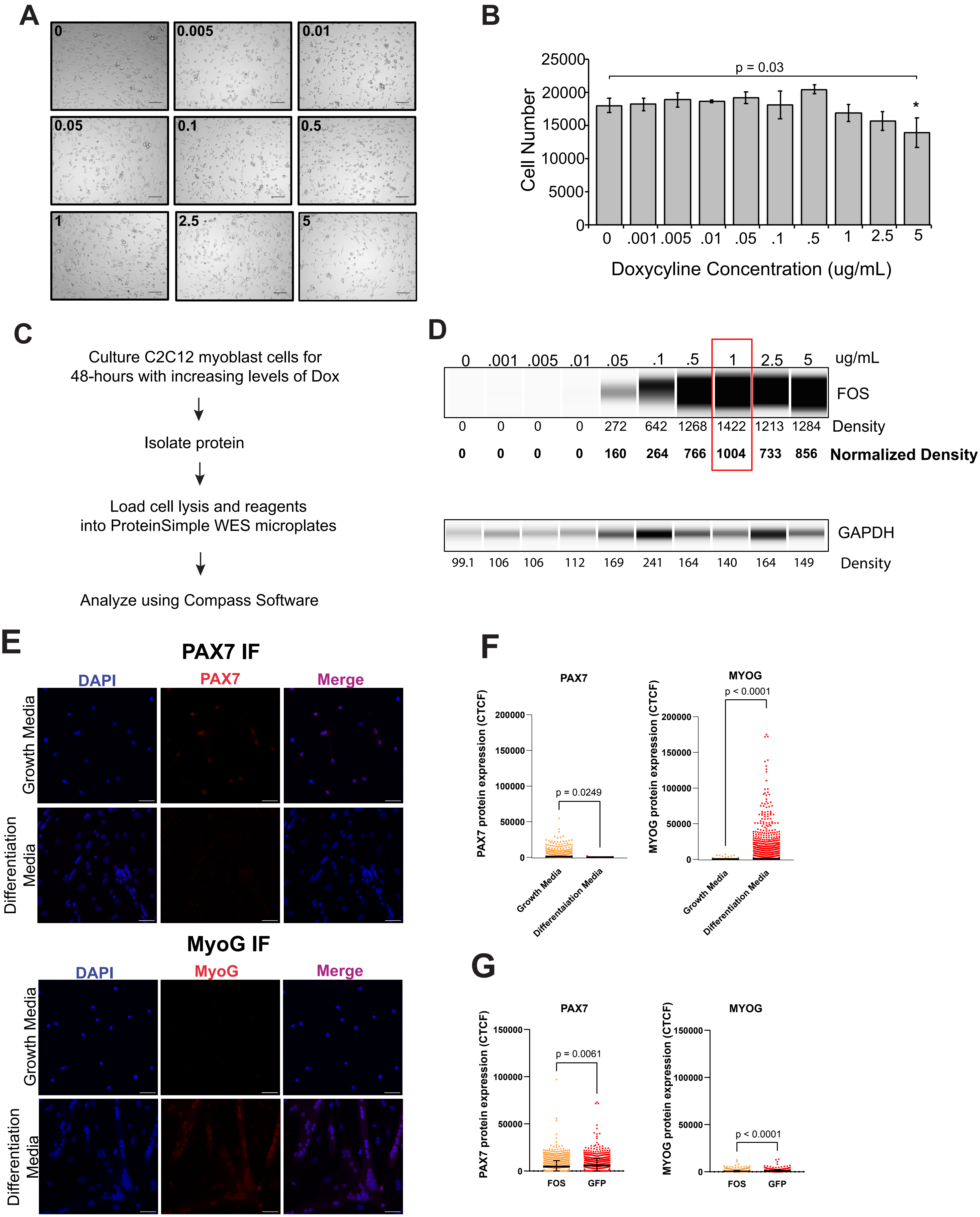

Supplementary Figure S2

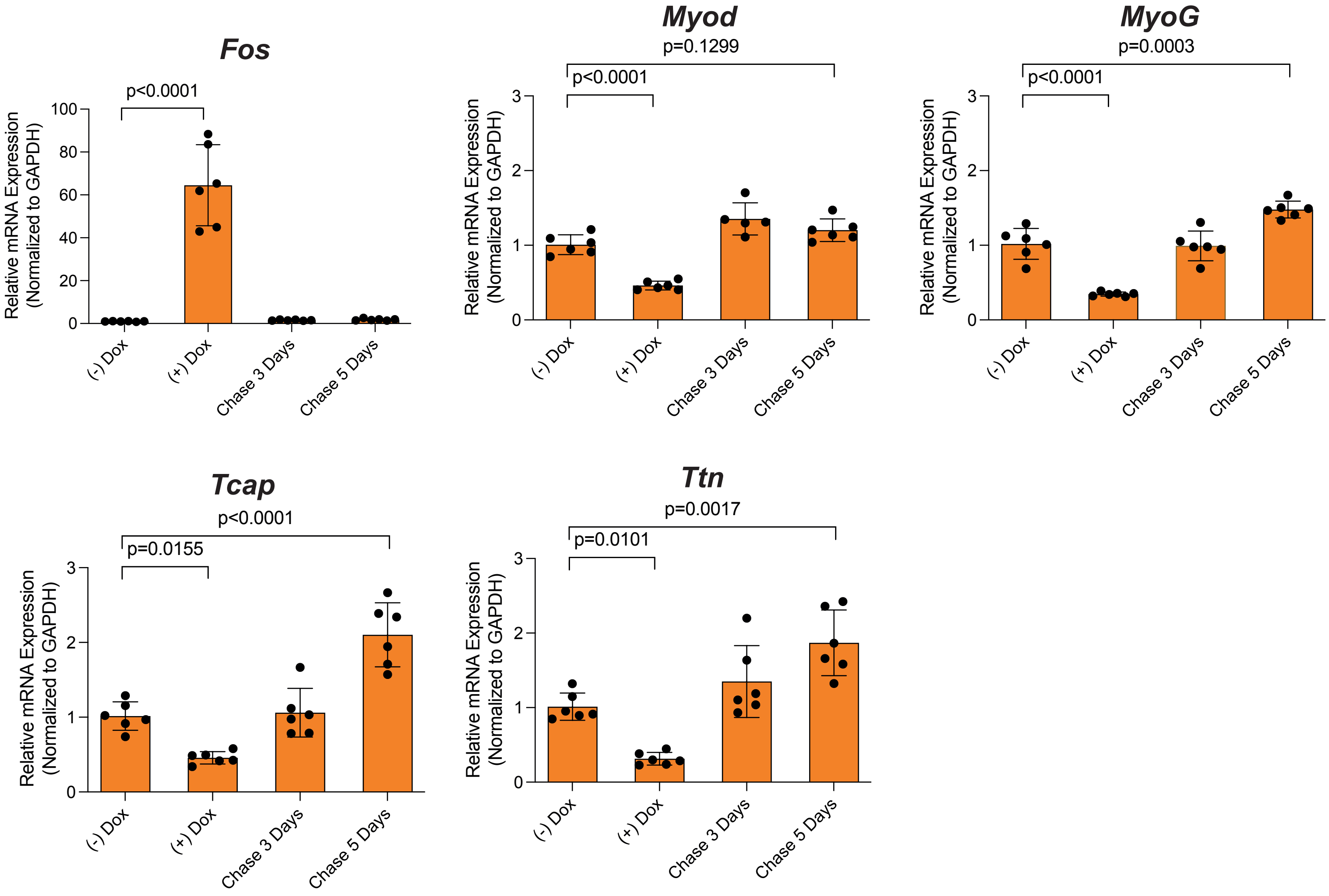

Supplementary Figure S3

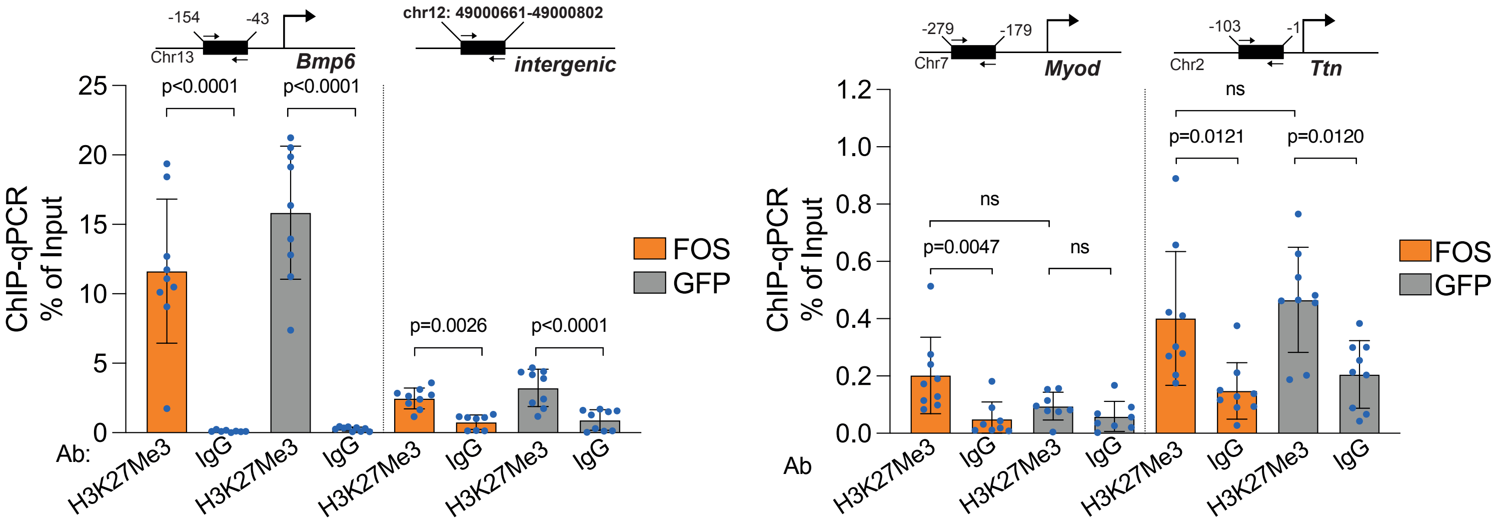

Supplementary Figure S4

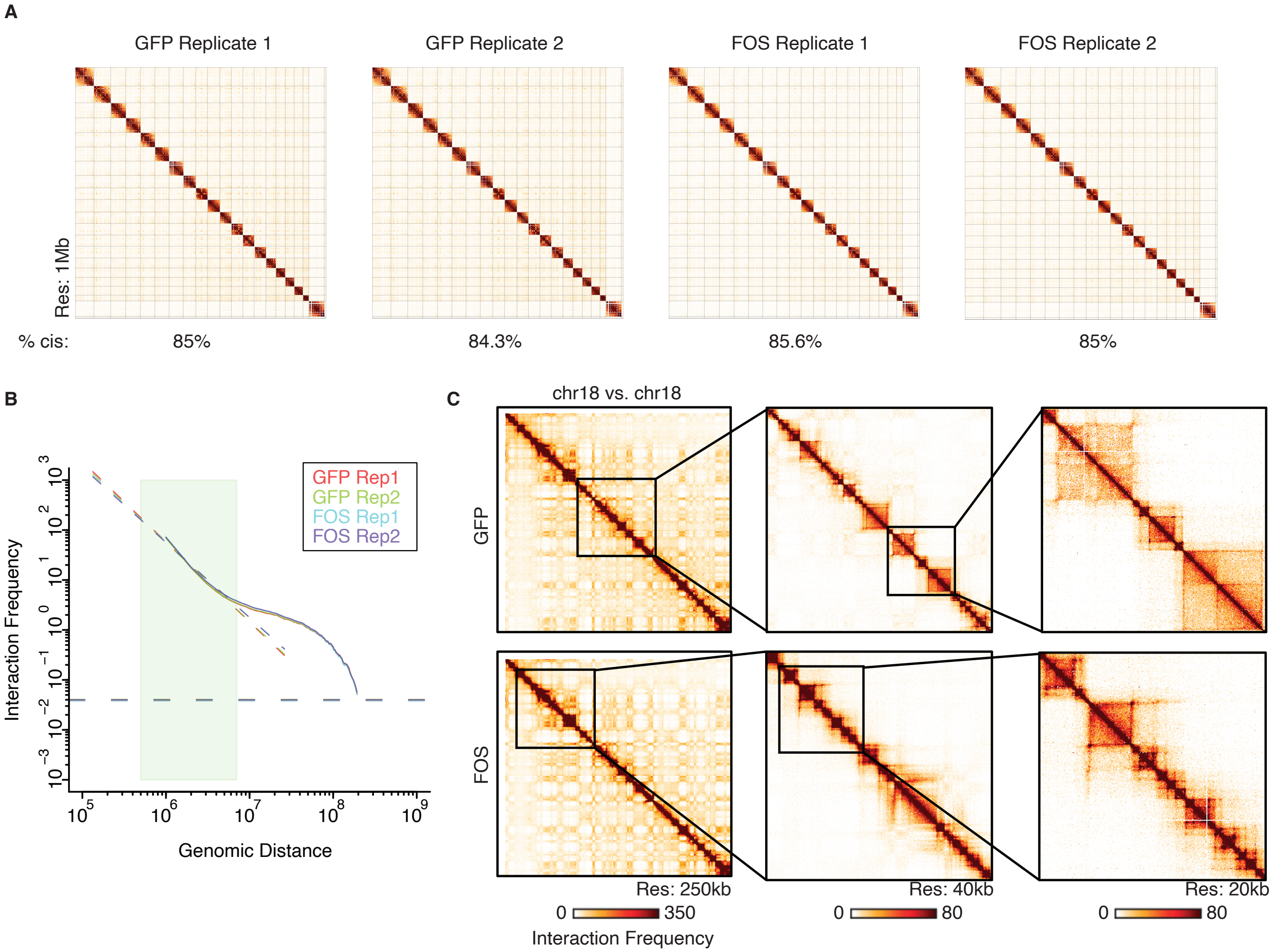

Supplementary Figure S5

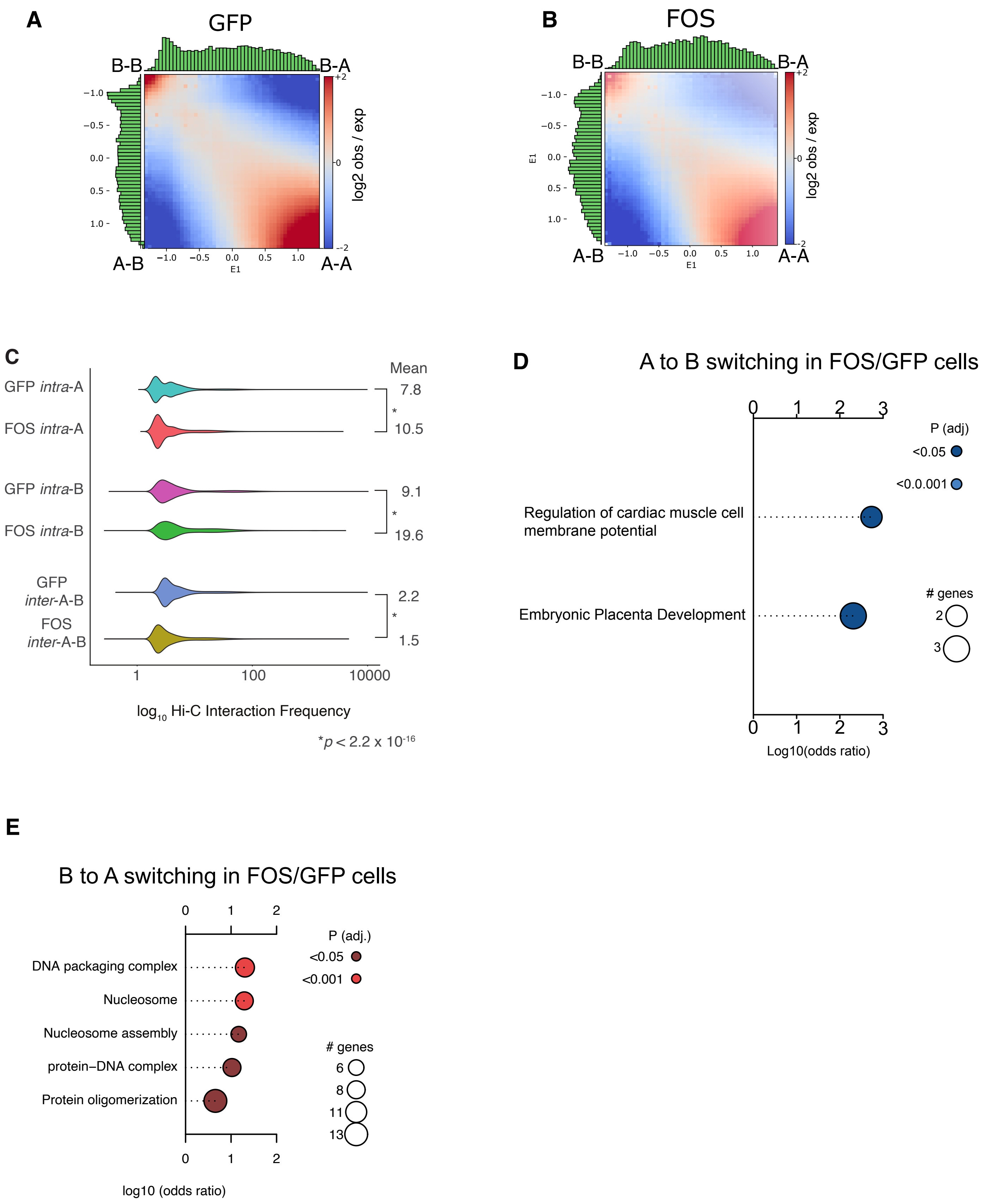

Supplementary Figure S6

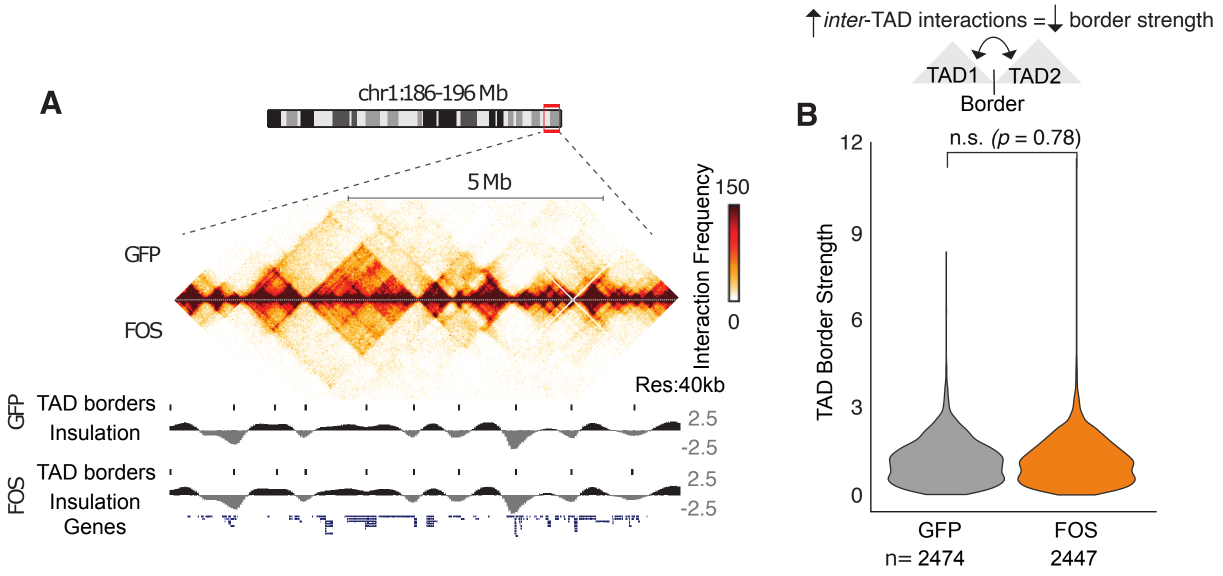

Supplementary Figure S7

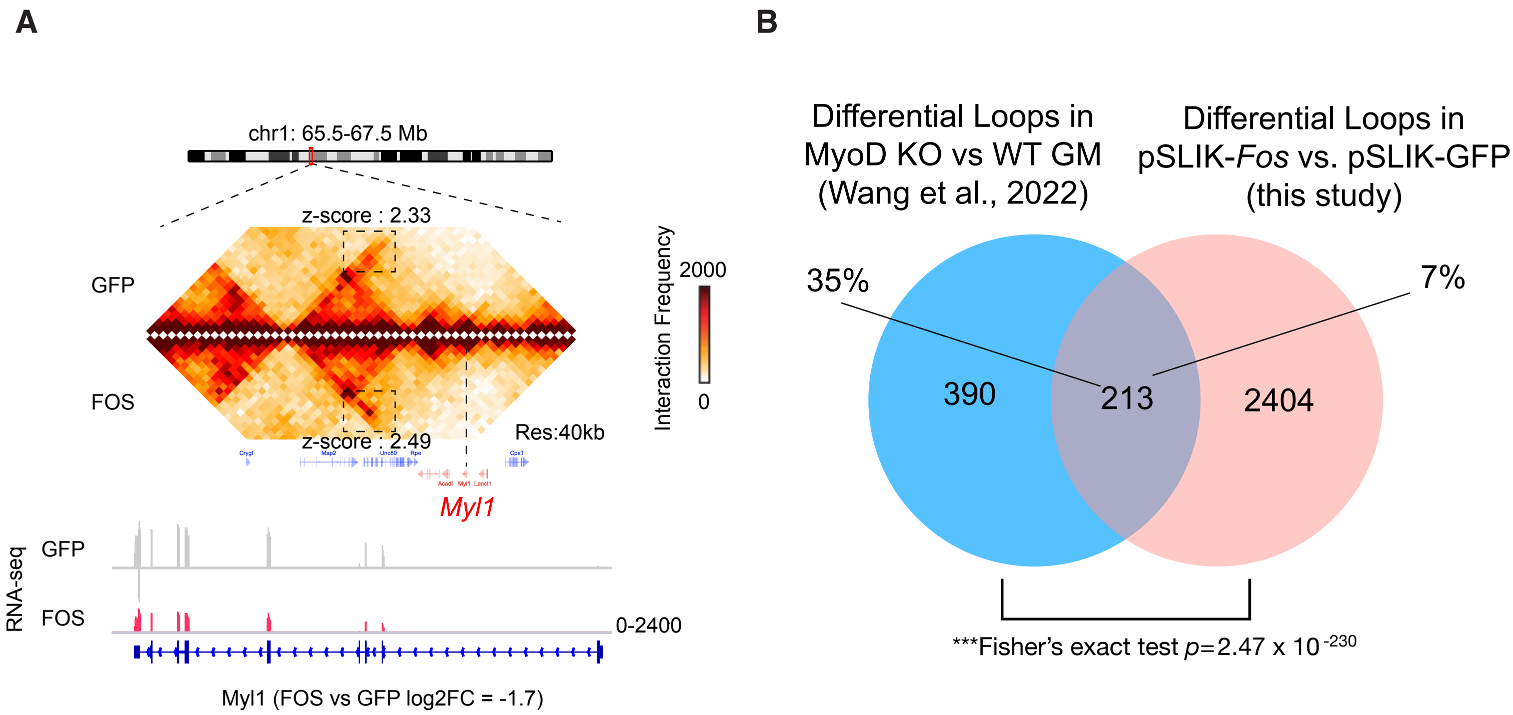

Supplement: Supplementary file 1 — Additional file 1: Supplementary Figure S1. Related to Figure 1. Validation and characterizing of our DOX-Inducible system for manipulating FOS expression in Muscle Progenitor Cells Ex Vivo. (A) Representative images of cultured muscle progenitor cells in GM supplemented with increasing amounts of Doxycycline (0, 0.005 μg/ml, 0.01 μg/ml, 0.05 μg/ml, 0.1 μg/ml, 0.5 μg/ml, 1 μg/ml, 2.5 μg/ml, 5 μg/ml) for 48 hours in culture. Scale bars represent 50 microns. (B) Quantification of the total number of Hoechst+ cells after 48 hours in GM supplemented with the indicated concentration of DOX (n=cells from 3mice). (C) Experimental Flowchart for detecting FOS protein (sc-7292) using ProteinSimple WES platform and analysis using the COMPASS software. (D) Virtual bands showing gradual increase of FOS protein with increasing amounts of DOX. Raw area of signal is shown for loading control (GAPDH) and FOS. FOS signal normalized to GAPDH is displayed, highlighting that 1 ug/ml of DOX was the lowest concentration that gave the maximal induction of FOS protein. (E) 20X images of two-week cultured muscle progenitor cells grown in GM for 48 hours or differentiated in DM for 72 hours and stained for PAX7 and MyoG. Nuclei stained with DAPI. Scale bar represents 50 microns. (F) Corrected total cell fluorescence (CTCF) for PAX7 (left) and MYOG (right) quantified in (E). n= 98-4184 cells (PAX7) and n= 2169-46118 cells (MYOG). (G) Corrected total cell fluorescence (CTCF) for PAX7 (left) and MYOG (right) in two-week cultured pSLIK-Fos and pSLIK-Gfp muscle progenitor cells. n=2295-2816 (PAX7) and n=1432-3779 (MYOG). Mean comparisons using One-way ANOVA with post-hoc Tukey test (B) and Mann Whitney U-test (F, G). Supplementary Figure S2. Related to Figure 3. FOS-dependent suppression of myogenic genes in muscle progenitor cells is reversible upon DOX removal. pSLIK-Fos cells were treated with 1 ug/ml DOX for 72-hours and then chased with media devoid of DOX for 3 and 5 days followed by RT-qPCR [file 13395_2022_303_MOESM1_ESM.pdf]
